# Molecular signalling towards mitochondrial breakdown is enhanced in skeletal muscle of patients with chronic obstructive pulmonary disease (COPD)

**DOI:** 10.1038/s41598-018-33471-2

**Published:** 2018-10-09

**Authors:** P. A. Leermakers, A. M. W. J. Schols, A. E. M. Kneppers, M. C. J. M. Kelders, C. C. de Theije, M. Lainscak, H. R. Gosker

**Affiliations:** 1 Department of Respiratory Medicine, NUTRIM School of Nutrition and Translational Research in Metabolism, Maastricht University Medical Centre+, Maastricht, The Netherlands; 2 Department of Cardiology, General Hospital Murska Sobota, Murska Sobota, Slovenia; 3 Faculty of Medicine, University of Ljubljana, Ljubljana, Slovenia

## Abstract

Loss of skeletal muscle mitochondrial oxidative capacity is well-established in patients with COPD, but the role of mitochondrial breakdown herein is largely unexplored. Currently, we studied if mitochondrial breakdown signalling is increased in skeletal muscle of COPD patients and associates with the loss of mitochondrial content, and whether it is affected in patients with iron deficiency (ID) or systemic inflammation. Therefore, mitophagy, autophagy, mitochondrial dynamics and content markers were analysed in *vastus lateralis* biopsies of COPD patients (N = 95, FEV_1_% predicted: 39.0 [31.0–53.6]) and healthy controls (N = 15, FEV_1_% predicted: 112.8 [107.5–125.5]). Sub-analyses were performed on patients stratified by ID or C-reactive protein (CRP). Compared with controls, COPD patients had lower muscle mitochondrial content, higher BNIP3L and lower FUNDC1 protein, and higher Parkin protein and gene-expression. BNIP3L and Parkin protein levels inversely correlated with mtDNA/gDNA ratio and FEV_1_% predicted. ID-COPD patients had lower BNIP3L protein and higher *BNIP3* gene-expression, while high CRP patients had higher BNIP3 and autophagy-related protein levels. In conclusion, our data indicates that mitochondrial breakdown signalling is increased in skeletal muscle of COPD patients, and is related to disease severity and loss of mitochondrial content. Moreover, systemic inflammation is associated with higher BNIP3 and autophagy-related protein levels.

## Introduction

Skeletal muscle weakness contributes to poor clinical outcome and is associated with increased morbidity and mortality in patients with chronic obstructive pulmonary disease (COPD)^1,2^. Important drivers of muscle weakness are the loss of skeletal muscle mitochondrial oxidative capacity and content, which, together with a oxidative to glycolytic fibre-type shift, are well-established in COPD^1,3,4^. Moreover, skeletal muscle mitochondria isolated from COPD patients were found to be functionally impaired and produce more reactive oxygen species, which indicates reduced mitochondrial health^5^.

Skeletal muscle oxidative capacity and mitochondrial quantity are mainly regulated by mitochondrial homeostasis, which is determined by the balance between mitochondrial biogenesis and mitochondrial breakdown^6^. Several studies have previously reported impaired skeletal muscle mitochondrial biogenesis regulation in COPD patients^3,7,8^, but data on mitochondrial breakdown is limited and its relation to the loss of oxidative capacity unknown.

Although individual mitochondrial proteins can be targeted for selective breakdown, bulk mitochondrial breakdown occurs via either autophagy-dependent (i.e. mitophagy) or autophagy-independent (i.e. mitochondria-derived vesicles (MDV)) lysosomal breakdown^9^. During mitophagy, mitochondria, or parts thereof, are separated from the mitochondrial network, engulfed by an autophagosomal membrane and subsequently broken down by a lysosome. This pathway can roughly be divided in receptor-mediated mitophagy, activated by mitophagy-receptors like BCL2/Adenovirus E1B 19 kDa protein-interacting protein 3 (BNIP3), BNIP3-like (BNIP3L), and FUN14 domain-containing protein 1 (FUNDC1)^10–12^, and PINK1/Parkin-mediated mitophagy, initiated by stabilization and activation of PTEN-induced putative kinase 1 (PINK1) and Parkin^13–15^. During MDV formation, which also requires PINK1 and Parkin, only a small portion of the mitochondrion is isolated from the mitochondrial network and targeted for lysosomal breakdown independently of autophagy^16,17^.

Mitochondria are highly dynamic organelles, which are constantly changing in size and shape. These changes are mediated through mitochondrial fission and fusion events. Mitochondrial fission has been suggested to be play an important role in isolating mitochondria from the mitochondrial network, priming them for mitophagy^9^. Fission is regulated by master-regulator Dynamin 1 Like (DNM1L), which is also known as Dynamin-related protein 1 (DRP1), and proteins like Mitochondrial fission 1 (FIS1), while fusion is mainly regulated on by Optic atrophy 1 (OPA1), and proteins from the Mitofusin (MFN) family^9^.

Interestingly, Guo *et al*. previously showed some indications of increased mitophagy in skeletal muscle of COPD patients^18^, but the relation between the mitochondrial breakdown and mitochondrial content in the skeletal muscle of COPD patients remains unstudied.

Although it is unlikely that the COPD-related lung pathology directly regulates the activation of these mitochondrial breakdown-pathways in skeletal muscle, these patients usually suffer from several extra-pulmonary manifestations which may be implicated in the development of skeletal muscle- and mitochondrial dysfunction, like cigarette smoke exposure, muscle inactivity, iron deficiency, and systemic inflammation^19–21^. Interestingly, both iron deficiency and systemic inflammation, which are commonly present in COPD patients, are not only linked to decreased exercise performance or oxidative capacity in chronic diseases^22,23^, but to induction of mitophagy in non-skeletal muscle models as well^24–26^. Therefore, it is likely that these manifestations have negative impact on the skeletal muscle mitochondrial content in COPD patients by inducing the initiation of mitochondrial breakdown

In the current study, we hypothesised that mitochondrial breakdown signalling is increased in skeletal muscle of COPD patients and is associated with the loss of mitochondrial content. In addition, we hypothesized that iron deficiency (ID) or enhanced systemic inflammation result in enhanced mitochondrial breakdown signalling in these patients. We tested these hypotheses by assessing differences in skeletal muscle expression of molecular markers of mitochondrial breakdown (i.e. BNIP3, BNIP3L, FUNDC1, PINK1, PARK2), general autophagy (i.e. LC3B, GABRARAPL1, SQSTM1, OPTN, CALCOCO2), and mitochondrial dynamics (i.e. DNM1L, FIS1, OPA1, MFN1) between COPD patients and controls, and studied if the mitochondrial breakdown markers correlated with markers for mitochondrial content (i.e. NDUFB8, SDHB, UQCR2, MT-COI, ATP5A, mtDNA/gDNA ratio). Moreover, we studied differences in expression of markers in these panels between COPD patients stratified by either iron deficiency, or the systemic inflammation marker C-reactive protein (CRP) plasma levels.

## Results

### COPD patients have altered levels of mitochondrial breakdown related markers compared with healthy controls

Patients had mild-to-severe COPD and were similar to control subjects with respect to age, sex, and body composition. In addition, patients had a different distribution of smoking status with a higher proportion of current- and ex-smokers compared with healthy controls. As expected, *MYH7* gene-expression, a surrogate marker for type I fibre proportion, was lower in the patients (Table 1).

**Table 1:** Characteristics of study population.

	Control	COPD
	(N = 15)	(N = 95)
**Demographics**		
Age, years^††^	65.1 (6.0)	65.0 (7.8)
Sex, % male^†^	60.0	66.3
Smoking status (never, ex, current), %^†^	47, 47, 6	0, 28, 72***
**Lung function**		
FEV_1_, % predicted^†††^	112.8 [107.5–125.5]	39.0 [31.0–53.6]***
FEV_1_/FVC, %^†††^	73.3 [69.3–77.8]	35.6 [28.1–46.4]***
GOLD stage (1, 2, 3, 4), %^†^		3, 23, 51, 23
**Body composition**		
BMI, kg/cm^2†††^	23.9 [23.0–26.1]	25.6 [22.2–27.3]
**Muscle composition**		
*MYH7* mRNA expression, Arbitrary Units^†††^	1.043 [0.602–1.265]	0.405 [0.166–0.693]***

Abbreviations: BMI, body mass index; FEV_1_, forced expiratory volume in 1 second; FVC, forced vital capacity; GOLD, Global Initiative for Chronic Obstructive Lung Disease. ^†^Data presented as percentage and statistical differences were calculated with chi-squared test; ^††^data presented as mean (SD) and statistical differences were calculated with independent samples t-test; ^†††^data presented as median [interquartile range] and statistical differences were calculated with Mann-Whitney U test. Statistical significance is depicted ***p < 0.001.

**Figure 1:**
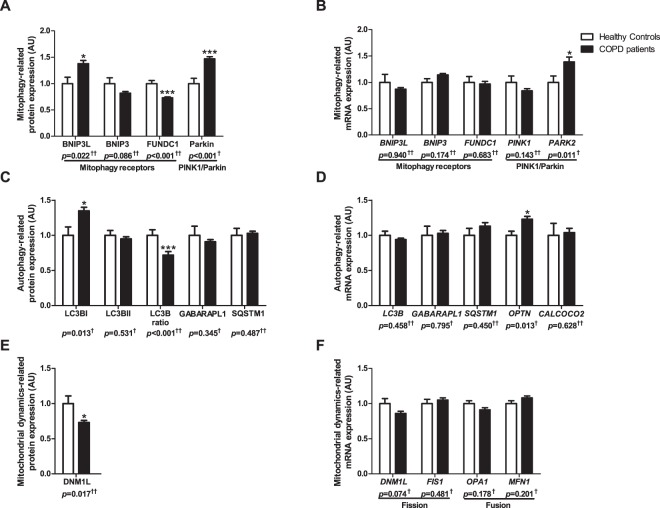
Protein and mRNA expression of markers related to mitophagy and autophagy in m. *vastus lateralis* of COPD patients and representative controls. Mitophagy-associated protein levels (**A**) and mRNA expression (**B**) are depicted. Autophagy-associated protein levels (**C**) and mRNA expression (**D**) are depicted. Mitochondrial dynamics-associated protein levels (**E**) and mRNA expression (**F**) are depicted. White bars represent healthy controls and black bars represent COPD patients. All samples derive from the same experiment and gels/blots were processed in parallel. Data presented as mean ± SEM. Variables had ^†^normal or ^††^non-normal distribution and *p-*value of ^†^parametric or ^††^non-parametric test and significant differences are depicted *p < 0.05, ***p < 0.001.

BNIP3L protein levels were higher in the *vastus lateralis* of COPD patients compared with controls (Fig. 1A). No differences were found for *BNIP3* and *BNIP3L* gene-expression (Fig. 1B). Moreover, FUNDC1 protein levels were found to be lower in the patients, while there was no corresponding change in *FUNDC1* gene-expression (Fig. 1A,B).

Microtubule associated protein 1A/1B-light chain 3B (LC3B) and γ-aminobutiric acid receptor-associated protein-like 1 (GABARAPL1) are autophagosomal membrane-based proteins with high affinity for respectively binding BNIP3/FUNDC1 and BNIP3L, which are needed for mitochondrial autophagosomal engulfment. Higher protein levels of the premature, cytoplasmic-based (LC3BI), but not of the mature, autophagosomal membrane-based (LC3BII), form of LC3B were found, resulting in a lower LC3BII/LC3BI protein ratio in COPD patients (Fig. 1C). Both protein levels and gene-expression of GABARAPL1, and gene-expression of *LC3B* were not different between groups (Fig. 1C,D).

Parkin protein- and gene-expression was higher in patients compared with controls (Fig. 1A,B), while gene-expression of PINK1 was not different (Fig. 1B). Although no differences were found in protein levels or gene-expression for the downstream autophagy-receptors sequestosome 1 (SQSTM1) and Calcium Binding And Coiled-Coil Domain 2 (*CALCOCO2*) (Fig. 1C,D), gene-expression of autophagy-receptor Optineurin (*OPTN*) was higher in COPD patients (Fig. 1D).

Protein levels of mitochondrial fission master regulator DNM1L were lower in *vastus lateralis* of COPD patients compared with controls (Fig. 1E). Gene-expression of mitochondrial fission or fusion markers was not different between the groups (Fig. 1F).

### COPD patients have a lower mitochondrial quantity compared with healthy controls, and both mitochondrial quantity and FEV_1_% predicted correlate moderately with the mitochondrial breakdown markers

**Figure 2:**
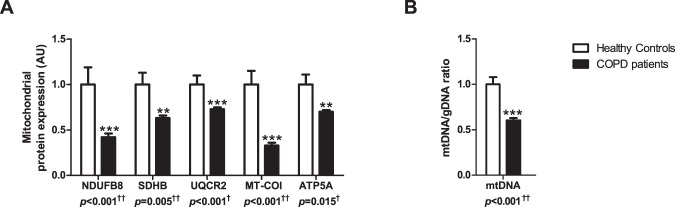
Protein levels of mitochondrial content markers and mtDNA/gDNA ratio in m. *vastus lateralis* of COPD patients and representative controls. Protein levels of different mitochondrial OXPHOS subunits are depicted (**A**). NDUFB8 as a subunit of OXPHOS complex I, SDHB of complex II, UQCRC2 of complex III, MT-COI of complex IV, and ATP5A of complex V. mtDNA/gDNA ratio is depicted (**B**). White bars represent healthy controls and black bars represent COPD patients. All samples derive from the same experiment and gels/blots were processed in parallel. Data presented as mean ± SEM. Variables had ^†^normal or ^††^non-normal distribution and *p-*value of ^†^parametric or ^††^non-parametric test and significant differences are depicted *p < 0.05, **p < 0.01, ***p < 0.001.

All tested subunits of oxidative phosphorylation (OXPHOS) complexes as well as the mtDNA/gDNA ratio were lower in the *vastus lateralis* of COPD patients compared with controls (Fig. 2A,B).

Since the mtDNA/gDNA ratio is widely accepted as a solid overall marker of mitochondrial content, we studied its correlation to the protein levels of BNIP3L (ρ = −0.288, *p* = 0.006), BNIP3 (ρ = 0.183, *p* = 0.083), FUNDC1 (ρ = 0.259, *p* = 0.014), and Parkin (ρ = −0.209, *p* = 0.049) (Supplementary Fig. 1A–D). The mtDNA/gDNA ratio correlated with FEV_1_% predicted (ρ = 0.407, *p* < 0.001) as well, suggesting a link between disease severity and mitochondrial content in COPD. Therefore, we also studied the correlation between FEV_1_% predicted and BNIP3L (ρ = −0.308, *p* = 0.003), BNIP3 (ρ = −0.003, *p* = 0.976), FUNDC1 (ρ = 0.461, *p* < 0.001) and Parkin (ρ = −0.361, *p* < 0.001) (Supplementary Fig. 2A–D).

### Iron deficient COPD patients have altered levels of BNIP3- and BNIP3L-mediated mitophagy markers compared with non-iron deficient COPD patients

To test whether muscle mitophagy is affected by iron deficiency in COPD patients, patients from the Golnik cohort were subdivided in ID-COPD and NID-COPD. Patients with ID-COPD were not different with respect to age, sex, smoking status, BMI, and plasma haemoglobin levels when compared with NID-COPD. However, they had a better lung function, and higher *MYH7* gene-expression (Table 2).

**Table 2:** Characteristics of the subset of COPD patients stratified by iron deficiency.

	non-iron deficient COPD	iron deficient COPD
	(N = 44)	(N = 19)
**Demographics**		
Age, years^††^	63.8 (8.4)	67.3 (8.8)
Sex, % male^†^	75.0	55.6
Smoking status (never, ex, current), %^†^	0, 12, 88	0, 6, 94
**Lung function**		
FEV_1_, % predicted^†††^	32.5 [25.3–40.3]	40.0 [34.0–60.0]*
FEV_1_/FVC, %^†††^	29.8 [26.5–37.8]	37.1 [33.0–43.5]*
GOLD (1, 2, 3, 4), %^†^	0, 7, 57, 36	0, 32, 53, 16*
**Body composition**		
BMI, kg/cm^2††^	24.7 (3.8)	25.9 (4.9)
**Iron status**		
Serum iron (μmol/L)^††^	19.6 (6.4)	14.6 (7.0)*
Transferrin (g/L)^†††^	2.4 [2.2–2.7]	2.5 [2.2–2.6]
Transferrin Saturation (%)^†††^	31.0 [24.0–40.0]	22.0 [17.0–26.0]**
Ferritin (ng/ml)^†††^	222 [158–294]	78 [58–116]***
Haemoglobin (g/l)^††^	147.0 (13.0)	142.1 (8.9)
**Muscle composition**		
*MYH7* mRNA expression, Arbitrary Units^†††^	0.179 [0.128–0.479]	0.517 [0.323–0.837]*

Abbreviations: BMI, body mass index; FEV_1_, forced expiratory volume in 1 second; FVC, forced vital capacity; GOLD, Global Initiative for Chronic Obstructive Lung Disease. ^†^Data presented as percentage and statistical differences were calculated with chi-squared test; ^††^data presented as mean (SD) and statistical differences were calculated with independent samples t-test; ^†††^data presented as median [interquartile range] and statistical differences were calculated with Mann-Whitney U test. Statistical significance is depicted *p < 0.05, **p < 0.01, ***p < 0.001.

**Figure 3:**
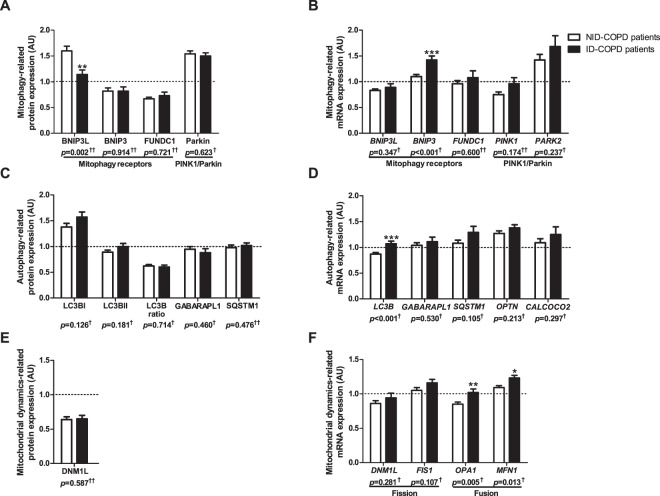
Protein and mRNA expression of markers related to mitophagy, autophagy, and mitochondrial dynamics in m. *vastus lateralis* of patients with NID-COPD and ID-COPD. Mitophagy-associated protein levels (**A**) and mRNA expression (**B**) are depicted. Autophagy-associated protein levels (**C**) and mRNA expression (**D**) are depicted. Mitochondrial dynamics-associated protein levels (**E**) and mRNA expression (**F**) are depicted. White bars represent healthy controls and black bars represent COPD patients. All samples derive from the same experiment and gels/blots were processed in parallel. Data presented as mean ± SEM. Dotted lines represent healthy controls. Variables had ^†^normal or ^††^non-normal distribution and *p-*value of ^†^parametric or ^††^non-parametric test and significant differences are depicted *p < 0.05, **p < 0.01, ***p < 0.001.

**Figure 4:**
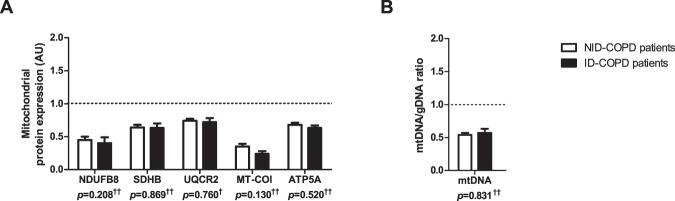
Protein levels of mitochondrial content markers and mtDNA/gDNA ratio in m. *vastus lateralis* of patients with NID-COPD and ID-COPD. Protein levels of different mitochondrial OXPHOS subunits are depicted (**A**). NDUFB8 as a subunit of OXPHOS complex I, SDHB of complex II, UQCRC2 of complex III, MT-COI of complex IV, and ATP5A of complex V. mtDNA/gDNA ratio is depicted (**B**). White bars represent non-iron deficient COPD patients and black bars represent iron deficient COPD patients. All samples derive from the same experiment and gels/blots were processed in parallel. Data presented as mean ± SEM. Dotted lines represent healthy controls. Variables had ^†^normal or ^††^non-normal distribution and *p-*value of ^†^parametric or ^††^non-parametric test.

BNIP3L protein levels were lower in the *vastus lateralis* of patients with ID-COPD compared with NID-COPD, while BNIP3 protein levels were not different (Fig. 3A). Although no changes in *BNIP3L* gene-expression were found, *BNIP3* gene-expression was higher in ID-COPD (Fig. 3B). No differences were found for FUNDC1, PINK1, or Parkin (Fig. 3A,B). Although *LC3B* gene-expression was higher in ID-COPD (Fig. 3D), there was no change in protein levels of LC3B or LC3BII/LC3BI ratio (Fig. 3C). None of the autophagy receptors downstream of PINK1/Parkin-mediated mitophagy (i.e. SQSTM1, OPTN, CALCOCO2) showed differential protein- or gene-expression (Fig. 3C,D). Interestingly, gene-expression of the mitochondrial fusion markers *OPA1* and *MFN1* was higher in ID-COPD, while no differences were found for the mitochondrial fission-related DNM1L and FIS1 on either protein or mRNA level (Fig. 3E,F). No differences in mitochondrial content were found in patients with ID-COPD compared with NID-COPD (Fig. 4A,B).

### Patients with high CRP have higher levels of BNIP3-mediated mitophagy markers compared with patients with low CRP

To test whether muscle mitophagy is affected by systemic inflammation in COPD patients, patients from the Maastricht cohort were divided in groups with high or low CRP. The groups were not different with respect to age, sex, smoking status, lung function, BMI, and *MYH7* gene-expression (Table 3).

**Table 3:** Characteristics of the subset of COPD patients stratified by CRP levels.

	low CRP (≤3.0 mg/L)	high CRP (>3.0 mg/L)
	(N = 19)	(N = 9)
**Demographics**		
Age, years^††^	65.1 (6.8)	66.1 (6.2)
Sex, % male^†^	47.0	67.0
Smoking status (never, ex, current), %^†^	0, 74, 26	0, 56, 44
**Lung function**		
FEV_1_, % predicted^†††^	62.6 [46.0–74.1]	47.9 [44.1–58.4]
FEV_1_/FVC, %^†††^	45.6 (13.3)	43.3 (6.4)
GOLD (1, 2, 3, 4), %^†^	16, 53, 32, 0	0, 22, 78, 0
**Body composition**		
BMI, kg/cm^2††^	24.8 (3.4)	26.7 (4.4)
**Systemic inflammation**		
CRP (mg/L)^†††^	1.1 [0.9–1.5]	7.7 [3.6–21.0]***
**Muscle composition**		
*MYH7* mRNA expression, Arbitrary Units^†††^	0.491 [0.410–1.125]	0.573 [0.349–1.075]

Abbreviations: BMI, body mass index; CRP, C-reactive protein; FEV_1_, forced expiratory volume in 1 second; FVC, forced vital capacity; GOLD, Global Initiative for Chronic Obstructive Lung Disease. ^†^Data presented as percentage and statistical differences were calculated with chi-squared test; ^††^data presented as mean (SD) and statistical differences were calculated with independent samples t-test; ^†††^data presented as median [interquartile range] and statistical differences were calculated with Mann-Whitney U test. Statistical significance is depicted ***p < 0.001.

**Figure 5:**
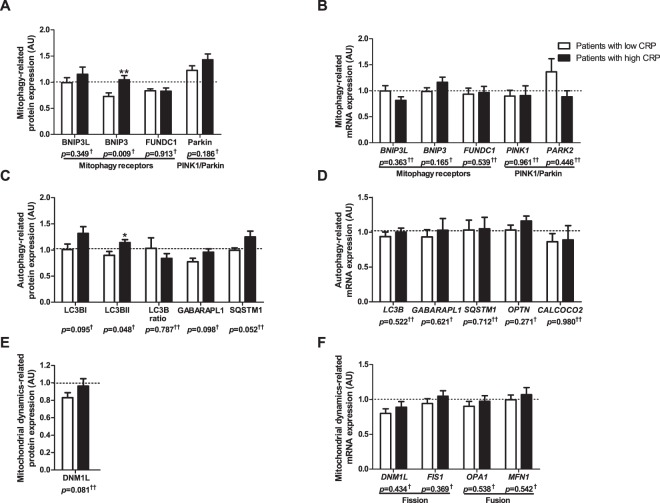
Protein and mRNA expression of markers related to mitophagy, autophagy, and mitochondrial dynamics in m. *vastus lateralis* of patients with low and high CRP. Mitophagy-associated protein levels (**A**) and mRNA expression (**B**) are depicted. Autophagy-associated protein levels (**C**) and mRNA expression (**D**) are depicted. Mitochondrial dynamics-associated protein levels (**E**) and mRNA expression (**F**) are depicted. White bars represent COPD patients with CRP ≤ 3.0 mg/L and black bars represent COPD patients with CRP** > **3 mg/L. All samples derive from the same experiment and gels/blots were processed in parallel. Data presented as mean ± SEM. Dotted lines represent healthy controls. Variables had ^†^normal or ^††^non-normal distribution and *p-*value of ^†^parametric or ^††^non-parametric test and significant differences are depicted *p < 0.05, **p < 0.01.

**Figure 6:**
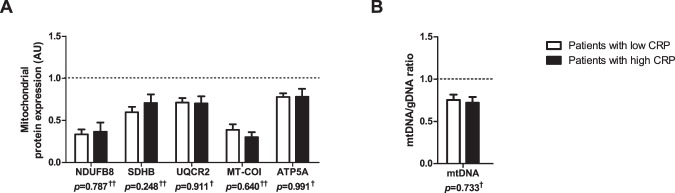
Protein levels of mitochondrial content markers and mtDNA/gDNA ratio in m. *vastus lateralis* of patients with low and high CRP. Protein levels of different mitochondrial OXPHOS subunits are depicted (**A**). NDUFB8 as a subunit of OXPHOS complex I, SDHB of complex II, UQCRC2 of complex III, MT-COI of complex IV, and ATP5A of complex V. mtDNA/gDNA ratio is depicted (**B**). White bars represent COPD patients with CRP ≤ 3.0 mg/L and black bars represent COPD patients with CRP** > **3 mg/L. All samples derive from the same experiment and gels/blots were processed in parallel. Data presented as mean ± SEM. Dotted lines represent healthy controls. Variables had ^†^normal or ^††^non-normal distribution and *p-*value of ^†^parametric or ^††^non-parametric test.

**Figure 7:**
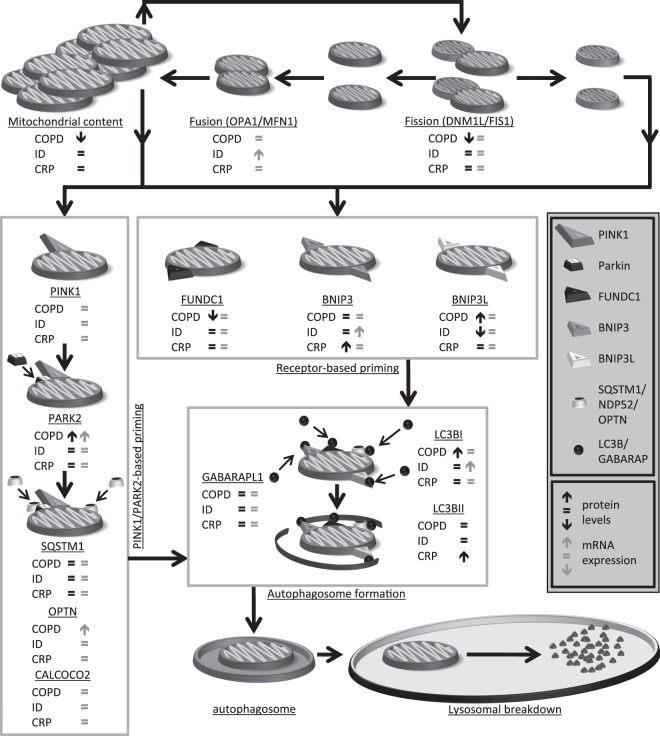
Schematic overview of differences in molecular signalling in the skeletal muscle of COPD compared with healthy control (COPD), ID-COPD compared with NID-COPD (ID) and COPD patients with high CRP compared with low CRP (CRP).

BNIP3 protein levels were higher in the *vastus lateralis* of patients with high CRP compared with patients with low CRP, while levels of other mitophagy-related proteins were not different (Fig. 5A). Interestingly, while LC3BII was the only measured autophagy-related protein which had significantly higher levels in the patients with high CRP, many other autophagy-related proteins showed similar trends (i.e. LC3BI, GABARAPL1, and SQSTM1) (Fig. 5C). Both mitophagy- and autophagy-related gene-expression was not different between the groups (Fig. 5B–D). No differences in any measured markers for mitochondrial dynamics were found (Fig. 5E,F), or mitochondrial content were found in patients with high compared with patients with low CRP (Fig. 6A,B). A schematic summary of all results is depicted in Fig. 7.

## Discussion

Our data indicates that mitochondrial breakdown is increased in quadriceps muscle of patients with COPD, illustrated by differential expression of both markers for receptor-mediated mitophagy and PINK1/Parkin-mediated mitochondrial breakdown. Secondly, we show that this expression pattern is associated with the decreased expression of mitochondrial content markers. Moreover, both BNIP3L and Parkin protein levels are inversely correlated with mitochondrial content and disease severity within the group of patients. Lastly, we report differences in the expression pattern of markers for BNIP3 and BNIP3L-mediated mitophagy in COPD patients suffering from either iron deficiency or systemic inflammation. Although the interpretation of this differential expression remains difficult for ID, it suggests increased BNIP3-mediated mitophagy signalling in patients with systemic inflammation.

Receptor-mediated mitophagy is described as a pathway targeting mitochondria for mitophagy based on activation of specific receptors by upstream signalling^10^. The currently reported higher protein levels of BNIP3L are a clear indication towards increased levels of this type of mitophagy in skeletal muscle of COPD patients. Moreover, BNIP3L was found to correlate inversely with the mtDNA/gDNA ratio and FEV_1_% predicted, suggesting a gradual increase in the level of BNIP3L-mediated mitophagy with increasing disease severity and loss of mitochondrial content. However, we observed a trend towards lower levels of BNIP3, which were previously found to be higher in skeletal muscle of COPD patients^18^. Since BNIP3 and BNIP3L are located on the outer mitochondrial membrane^27–29^, an increase in BNIP3 protein expression might be masked by the lower mitochondrial content in our COPD population.

FUNDC1 is a relatively new hypoxia-regulated mitophagy-receptor of which the activity is dependent on its phosphorylation state rather than its transcriptional regulation. The activation of FUNDC1 therefore results in decreased protein levels as a direct result of increased mitophagy^30–32^. Indeed, we found lower FUNDC1 protein levels in COPD patients, which were correlated to both the mtDNA/gDNA ratio as well as the FEV_1_% predicted, which could indicate disease severity dependent increased FUNDC1-mediated mitophagy in patients. However, FUNDC1 is a mitochondrial membrane-based protein as well, and lower FUNDC1 levels might therefore be directly caused by the lower mitochondrial quantity in COPD patients. Although acute hypoxic stress could also result in a decrease of FUNDC1 protein independently of mitophagy^33^, this is unlikely to be the case in our population due to the chronic and stable disease stage in which we obtained the biopsies.

PINK1 and Parkin have been described as major players in the mitophagy pathway, priming dysfunctional mitochondria for autophagosomal-lysosomal. Although the regulation and signal-transduction of PINK1 and Parkin is very complex, current literature suggest that PINK1 functions as an initiation protein and Parkin as a signal-amplifying protein in the priming of mitochondria for mitophagy^13,15^. In addition to mitophagy, both PINK1 and Parkin have been associated with MDV formation, an autophagy-independent mitochondrial breakdown pathway^16^. We currently report higher gene-expression and protein levels of Parkin, suggesting increased Parkin-mediated mitochondrial breakdown in COPD patients. Taken together, these data, which are in concert with data from a previous study^18^, indicate increased mitochondrial priming for lysosomal breakdown via either mitophagy or MDVs. Moreover, since Parkin was found to correlate inversely with mtDNA/gDNA ratio and the FEV_1_% predicted, the increase in Parkin protein levels appears to be more pronounced in both more severe patients and in patients with increased loss of mitochondrial content.

While DNM1L–mediated mitochondrial fission has been described as a prerequisite for mitophagy, MDV biogenesis could be performed independently of DNM1L^9,16,34,35^. Mitochondrial fission and fusion regulation is complex and highly integrated in muscle mass and quality control pathways^9^. Although we did not quantify actual mitochondrial fission, the reported lower DNM1L protein-, and trend towards lower gene-expression, suggests that mitochondrial fission regulation is marginally decreased in COPD patients. Interestingly, a previous study reported decreased mitochondrial numbers while mitochondrial size was unaltered in the *vastus lateralis* of COPD patients^36^, suggesting unaltered mitochondrial dynamics in these patients. Although we did not find a correlation between DNM1L protein levels and mtDNA/gDNA ratio in the current cohort of COPD patients (ρ = 0.108, *p* = 0.312), the lower DNM1L levels could be part of a physiological response to maintain the amount of DNM1L per mitochondrion. Together with the current literature, our results are urging caution with interpretation of fission and fusion related signalling data.

The currently observed lower levels of mitochondrial content markers are in line with the well-established loss of oxidative capacity in skeletal muscle of patients with COPD^2,3,5,37^, as well as with the well-described oxidative to glycolytic fibre-type shift, which is consistently observed in the peripheral skeletal muscle of COPD patients^4^. Although we did not determine the percentage of oxidative type I fibres immunohistochemically in our study population, the lower expression of the *MYH7* gene (a surrogate marker for type I fibres as it encodes the type I myosin heavy chain isoform^38^) in COPD is indicative of a decreased oxidative fibre type percentage which is in line with literature. Although we cannot exclude that differences in fibre type distribution influenced our findings, the fact that we found increased levels of mitophagy-related signalling despite the lower MYH7 gene expression, indicative of less mitochondria-rich type I fibres, in the patients suggests that enhanced mitochondrial breakdown may indeed be involved in the loss of muscle oxidative capacity in COPD.

Interestingly, mtDNA/gDNA ratio was correlated, or showed a trend towards correlation, with BNIP3L, BNIP3, FUNDC1, and Parkin. Furthermore, mtDNA/gDNA ratio was also correlated with FEV_1_% predicted, which in turn was correlated with BNIP3L, FUNDC1 and Parkin. Together, these correlations show the highly interconnected nature of mitochondrial breakdown signalling, mitochondrial content, and disease severity.

Since it is unlikely that the COPD-related lung pathology directly regulates the activation of mitochondrial breakdown-pathways in skeletal muscle, we previously suggested a role for extra-pulmonary systemic manifestations^19^. We therefore studied if iron deficiency and systemic inflammation are involved in the mitochondrial breakdown regulation in our COPD population.

Iron deficiency, often present but disregarded in COPD patients, has recently been shown to initiate mitophagy in non-skeletal muscle based models^24,39^. Unexpectedly, our ID-COPD patients had lower skeletal muscle BNIP3L protein levels than NID-COPD patients, resembling the levels of healthy controls, which could indicate decreased rather than increased mitophagy. However, ID-COPD patients had higher *BNIP3* and *LC3B* gene- but not protein-expression, which could also indicate increased turnover of BNIP3 due to increased mitophagy. No differences were found in markers of either the FUNDC1- or PINK1/Parkin-mediated mitochondrial breakdown in patients stratified by iron status. This is in line with a previous report showing iron depletion results in PINK1/Parkin-independent mitophagy in neuroblast cells^24^. In conclusion, iron deficiency results in differential expression of BNIP3 and BNIP3L-related mitophagy markers, but does not seem to result in increased overall mitophagy in ID-COPD in our study population. Moreover, both the currently reported higher *MYH7* gene-expression, indicative of more oxidative type I fibres, and better lung function in ID-COPD patients might affect our results, and therefore further research is needed to elucidate the exact impact of iron deficiency on mitophagy and the skeletal muscle oxidative phenotype.

The mitophagy-receptors BNIP3 and BNIP3L are known to be under transcriptional control of HIF-1α^40^, which is potentially regulated by several COPD-associated manifestations such as inflammation^41^ and hypoxia^42^. Indeed, patients with high CRP had higher skeletal muscle BNIP3 protein levels combined with higher levels of down-stream autophagy-related proteins. Although previous reports have indicated PINK1 and Parkin as important players in inflammation-mediated mitophagy both *in vitro* and *in vivo*, we report no differences in markers of either the FUNDC1- or PINK1/Parkin-mediated mitochondrial breakdown in patients with high CRP^25,26^. However, these studies were performed with models exposed to profound inflammatory stimuli, almost certainly exceeding the low-grade systemic inflammation present in our patients, and might therefore surpass clinical relevance for our study population. In conclusion, we report higher BNIP3 and autophagy-related protein levels in patients with high systemic inflammation.

Besides iron deficiency and systemic inflammation, smoke exposure and increased muscle inactivity are among COPD-related extra-pulmonary manifestations which could potentially affect skeletal muscle mitochondrial impairments^19,20^. Indeed, our population of COPD patients had more current- and ex-smokers compared with the control population. Moreover, it is highly likely that these patients had lower physical activity levels as well^1^, albeit that available literature in which physical activity levels have been linked to muscle biopsy analyses in COPD suggest that inactivity is a poor determinant of the loss of muscle oxidative phenotype in this disorder^3,43,44^. Although these manifestations were not the focus of the current study, it cannot be excluded that these factors contributed to the currently observed modulations in mitochondrial breakdown signalling in COPD patients and therefore these manifestations might be interesting targets for future research.

Although we show clear differences in mitochondrial breakdown-related markers, some limitations have to be addressed. First, we did not fully quantify the process of mitochondrial breakdown itself. This would require measuring actual mitophagy and/or MDV flux, for example by repeated measurements while blocking lysosomal breakdown^45^. This is not feasible in human studies however, since lysosomal breakdown is required for long-term muscle maintenance. Moreover, continuous assessment of the studied parameters throughout disease progression would have been favourable, enabling determination of both the chronological order and the fluctuation of the studied processes. However, due to the invasive nature of muscle biopsies, and since they represent only one specific moment in time, we chose to study one time-point in a large heterogeneous group of patients.

In conclusion, this study shows altered expression of molecular markers for pathways of mitochondrial breakdown in skeletal muscle of COPD patients, which are related to disease severity and loss of mitochondrial quantity. Moreover, we report that both systemic inflammation and iron deficiency are associated with alterations in molecular markers for BNIP3 and BNIP3L-mediated mitophagy, and that while we report no coherent changes for ID-COPD, our results show higher BNIP3 and autophagy-related protein levels in patients with increased systemic inflammation. Taken together, the current data supports a potential role for mitochondrial breakdown underlying the loss of mitochondrial content in skeletal muscle of COPD patients, and identifies systemic inflammation as a possible mitophagy-inducing manifestation. This data is instrumental in understanding disturbed mitochondrial homeostasis in skeletal muscle of COPD, potentially leading towards new targets for maintaining or enhancing mitochondrial health.

## Methods

### Ethical approval

*Vastus lateralis* biopsies of COPD patients and healthy controls, previously gathered in two different cohort studies (Maastricht cohort; www.trialregister.nl: NTR1402, and Golnik cohort; www.clinicaltrials.gov: NCT02550808) were analysed post-hoc. The Maastricht cohort study was approved by the Maastricht University Medical Centre+ ethical review board (Maastricht, the Netherlands), and the Golnik cohort study was approved by the Slovenian National Medical Ethics Committee (Ljubljana, Slovenia). In total the studied population consisted of 95 COPD patients with clinically stable disease (i.e. free from exacerbations in the 4 weeks prior to the study) and 15 healthy age-matched controls. Study protocols were in accordance with the latest version of the Helsinki Declaration, approved by the respective ethics committees, and written informed consent was obtained from all subjects prior to the start. Spirometry was performed according to the European Respiratory Society guidelines. Data including smoking status, calculated FEV_1_ percentage predicted (FEV_1_% predicted)^46^ and disease severity, based on Global Initiative for Chronic Obstructive Lung Disease (GOLD) stage^47^, was available for all patients.

Muscle biopsies were obtained under local anaesthetic under resting conditions using the needle biopsy technique, snap-frozen in liquid nitrogen, and stored at −80 °C until molecular analyses as described previously^3,48^.

### mRNA extraction and quantification

mRNA extraction and quantification was performed as described previously^49^ with primer details shown in Supplementary Table 1. In short, tissue was homogenized with the Beat-Beater in presence of TRI-reagent (Sigma-Aldrich, Zwijndrecht, the Netherlands), and RNA was isolated by TRI-reagent/Chloroform extraction and subsequently precipitated from the aqueous phase using glycogen-containing isopropanol. RNA concentrations were measured spectrophotometrically using a Nanodrop UV-Vis spectrophotometer (Thermo Scientific, Landsmeer, The Netherlands). 400 ng RNA was diluted in nuclease free H_2_O and reverse transcribed to cDNA using the Tetro cDNA synthesis kit (Bioline, Waddinxveen, The Netherlands) according to the manufacturer’s instructions. qPCR reactions contained Sensimix SYBR & ROX (Bioline, Waddinxveen, The Netherlands) and primer mix and were run in a 384 well white opaque plate on a LightCycler 480 system (Roche, Almere, The Netherlands). Melting curves were analysed to verify specificity of the amplification, and relative quantity of the targets was assessed by LinRegPCR software (v2014.8.1). Three reference genes (RPLP0, B2M, and PPIA) were used to calculate a GeNorm correction factor, which was used to normalize expression of the target genes. Specific sample measurements were excluded when individual PCR efficiency was deviating from average PCR efficiency.

### DNA extraction and quantification

DNA was purified from the organic fraction, generated with the TRI-reagent/Chloroform RNA extraction, according to manufacturer’s protocol (Sigma-Aldrich, Zwijndrecht, the Netherlands), with the modification of centrifugation speed to 12,000 g. DNA was pelleted, and dissolved in TE buffer (Sigma-Aldrich, Zwijndrecht, The Netherlands). qPCR was performed as described previously^49^ with mitochondrial (COX-II) and genomic (RPL13A) specific primers (Supplementary Table 1). Data is presented as ratio of the relative copy number of mtDNA over gDNA.

### Western Blot

Western Blotting and quantification was performed as described previously^49^, with primary antibodies listed in Supplementary Table 2. In short, tissue was homogenized in 600 μl of Immunoprecipitation lysis buffer (50 mM Tris, 150 mM NaCl, 10% glycerol, 0.5% Nonidet P40, protease and phosphatase inhibitors (Roche, Almere, The Netherlands)) with a Polytron homogenizer (Kinematica, Eschbach, Germany) and centrifuged at 14,000 g at 4 °C for 30 min. 5 μg of either unheated or heated (5 min at 100 °C) protein in 1x Laemmli sample buffer (0.25 M Tris-HCL ph6.8; 8% (w/v) SDS; 40% (v/v) glycerol; 0.4 M DTT and 0.02% (w/v) Bromophenol Blue) was separated on a Criterion XT Precast 4–12 or 12% Bis-Tris gel (Bio-Rad Laboratories B.V., Veenendaal, The Netherlands) in XT MOPS or MES running buffer (Bio-Rad Laboratories B.V., Veenendaal, The Netherlands) by gel electrophoresis. Proteins were transferred to a nitrocellulose membrane (Bio-Rad Laboratories B.V., Veenendaal, The Netherlands) by electroblotting at 100 V for 60 min in transfer buffer (25 mM Tris, 192 mM Glycine, 20% (vol/vol) methanol).

Membranes were stained with 0.2% PonceauS in 1% acetic acid (Sigma-Aldrich, Zwijndrecht, The Netherlands) and imaged with the Amersham™ Imager 600 (GE Healthcare Life Sciences, Eindhoven, The Netherlands), to correct for protein loading. Subsequently, the membranes were blocked at room temperature (RT) in Tween20 Tris-buffered saline (TBST; 20 mM Tris, 137 mM NaCl, 0.1% (vol/vol) Tween20, pH 7.6) containing 3% (w/v) nonfat dry milk (Campina, Zaltbommel, The Netherlands), washed in TBST, and incubated overnight with primary antibody diluted 1:500–1:1,000 in TBST with 3% (w/v/) BSA or non-fat dry milk at 4 °C. Membranes were washed, incubated with a HRP-conjugated secondary antibody (Vector Laboratories, Amsterdam, The Netherlands), diluted 1:10,000 in 3% (w/v) non-fat dry milk in TBST, washed, incubated in Supersignal West PICO or FEMTO Chemiluminescent Substrate (Thermo Scientific, Landsmeer, The Netherlands), and imaged using the Amersham™ Imager 600. Images were quantified with Image Quant software (GE Healthcare Life Sciences, Eindhoven, The Netherlands). Samples and protein loading reference lanes were randomly distributed within and between multiple gels (Supplementary Fig. 3). All samples derive from the same experiment and gels/blots were processed in parallel. Target protein quantity was corrected for total protein content, and between-gel differences, and presented as a fold change relative to the control group. Measurements were excluded when signs of protein deterioration were found. All original data is included in Supplementary Figs 4–13.

### Blood measurements

Blood samples were obtained from all patients at the start of the study protocol. Serum ferritin levels, transferrin saturation, iron levels, and haemoglobin levels were measured in the patients from the Golnik cohort. Subjects were considered iron deficient (ID-COPD) when either absolute iron deficiency (ferritin <100 ng/ml) or functional iron deficiency (ferritin 100–300 ng/ml and transferrin saturation <20%) was present^22^. Other patients were defined as non-iron deficient (NID-COPD) patients

Serum CRP was determined as a marker for systemic inflammation with the CardioPhase® high-sensitive CRP kit (Siemens Healthcare Diagnostics Inc., Newark, USA) with a lower limit of detection of 0.18 mg/L, in the COPD patients from the Maastricht cohort^50^. Subjects were considered to have low CRP when CRP ≤ 3.0 mg/L and high CRP when CRP > 3.0 mg/L.

### Statistics

Variables were tested for normality using Shapiro-Wilk Test. Patient characteristics were presented in tables as percentage, mean (SD), or median [interquartile range] based on distribution of data. All molecular markers were, independently of normal distribution, presented graphically in bar charts as mean + SEM for coherence and clarity purposes. Statistical relevance of observed group-differences was tested with chi-squared test for categorized variables, Mann-Whitney U test for non-normal distributed variables, or independent *t-*test for normal distributed variables. The exact number of cases tested per variable is depicted in the tables for patient characteristics, and in Supplementary Table 1 and 2 for molecular markers. Correlations were tested with Spearman’s rho analysis’s in the combined cohorts (N = 89–94). All statistical tests were computed using IBM SPSS Statistics software (version 22.0, IBM Corp., Armonk, NY, USA).

## Electronic supplementary material

Supplementary material and methods

## Data Availability

All Western Blot Raw data generated or analysed during this study are included in this published article (and its Supplementary Information files). Other datasets generated during and/or analysed during the current study are available from the corresponding author on reasonable request.
