# Supplementary material for: Molecular signalling towards mitochondrial breakdown is enhanced in skeletal muscle of patients with chronic obstructive pulmonary disease (COPD)
Source: Sci Rep. 2018 Oct 9;8:15007. doi: 10.1038/s41598-018-33471-2 (PMC6177478; doi:10.1038/s41598-018-33471-2)
Supplement: Supplementary file 1 — Supplementary material and methods [file 41598_2018_33471_MOESM1_ESM.docx]

# Title Molecular signalling towards mitochondrial breakdown is enhanced in skeletal muscle of patients with chronic obstructive pulmonary disease (COPD)

# Authors

P.A. Leermakers^1^, A.M.W.J. Schols^1^, A.E.M. Kneppers^1^, M.C.J.M. Kelders^1^, C.C. de Theije^1^, M. Lainscak^2,3,#^, H.R. Gosker^1,#^

^#^ Both authors contributed equally

**Supplementary material**

# Supplementary Results


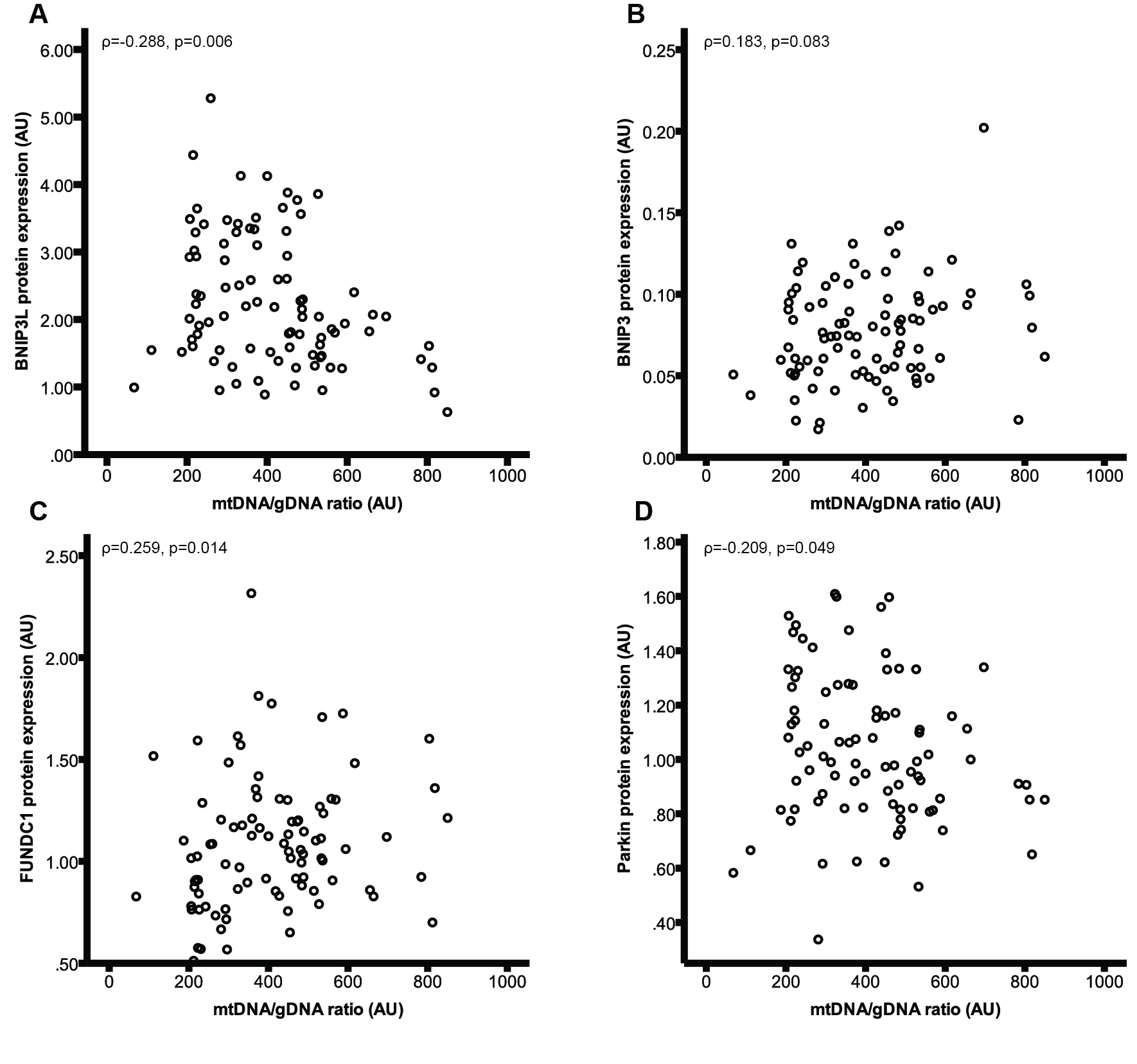


**Supplementary Figure 1: Correlations of mitophagy markers with mtDNA/gDNA ratio**. Correlations of mtDNA/gDNA ratio with BNIP3L (**A**), BNIP3 (**B**), FUNDC1 (**C**), and Parkin (**D**), are depicted in the group of COPD patients (N=89-91).


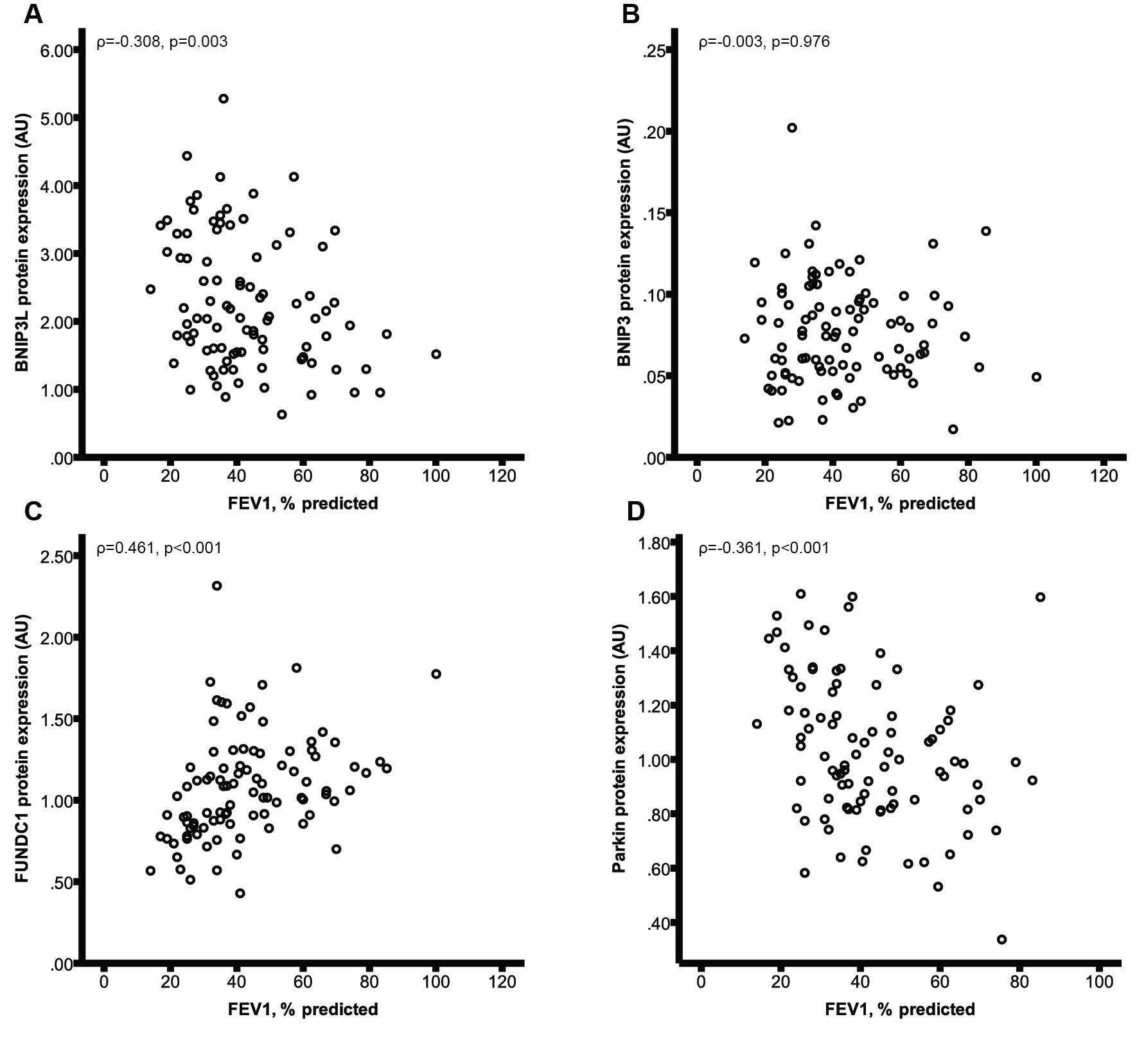


**Supplementary Figure 2: Correlations of mitophagy markers with FEV_1_%**. Correlations of FEV_1_% with BNIP3L (**A**), BNIP3 (**B**), FUNDC1 (**C**), and Parkin (**D**), are depicted in the group of COPD patients (N=92-94).

# Supplementary Methods

**mRNA and DNA extraction and quantification**

Supplementary Table 1: Primer sequences for qPCR. Target gene, sense primer, antisense primer, Ensembl identifier, and specific number of samples per analysis are depicted for each target.

| **Target** | **Sense primer** | **Antisense primer** | **Ensembl identifier** | **N**^†^ | **N**^††^ | **N**^†††^ |
| --- | --- | --- | --- | --- | --- | --- |
| ***RPLP0*** | TCTACAACCCTGAAGTGCTTGATATC | GCAGACAGACACTGGCAACATT | ENSG00000089157 |  |  |  |
| ***B2M*** | CATCTGCACTGCCAAGACTGA | TTCATGCCTTCTTTCACTTTGC | ENSG00000196262 |  |  |  |
| ***PPIA*** | CTGTGCTCGCGCTACTCTCTCTT | TGAGTAAACCTGAATCTTTGGAGTACGC | ENSG00000166710 |  |  |  |
| ***RPL13A*** | CCTGGAGGAGAAGAGGAAAGAGA | TTGAGGACCTCTGTGTATTTGTCAA | ENSG00000142541 |  |  |  |
| ***MYH7*** | CCTGGAACATCTGGAGACCT | AGCTGCTTTCGGACCTTCT | ENSG00000092054 | 15;93 | 43;18 | 19;9 |
| ***BNIP3*** | AGCGCCCGGGATGCA | CCCGTTCCCATTATTGCTGAA | ENSG00000176171 | 15;93 | 43;18 | 19;9 |
| ***BNIP3L*** | CTGCGAGGAAAATGAGCAGTCTCT | GCCCCCCATTTTTCCCATTG | ENSG00000104765 | 15;93 | 43;18 | 19;9 |
| ***FUNDC1*** | GAAACGAGCGAACAAAGCAG | GCAAAAAGCCTCCCACAAAT | ENSG00000069509 | 15;92 | 42;18 | 19;9 |
| ***PINK1*** | CGTTGGACACGAGACGCTTGCA | GCAATGTAGGCATGGTGGCTTCATAC | ENSG00000158828 | 15;93 | 43;18 | 19;9 |
| ***PARK2*** | GGTTTGCCTTCTGCCGGGAATG | CTTTCATCGACTCTGTAGGCCTG | ENSG00000185345 | 15;92 | 42;18 | 19;9 |
| ***OPTN*** | AAGGAGCAACTGGCATTGCA | TCTCCATCAAGGACTGCCTG | ENSG00000123240 | 15;92 | 42;18 | 19;9 |
| ***CALCOCO2*** | TCTTTGCCGTATCAAGTACCTACT | GGCATTTCTTGATGGAGAGCG | ENSG00000136436 | 15;92 | 42;18 | 19;9 |
| ***LC3B*** | ACCATGCCGTCGGAGAAGAC | TCTCGAATAAGTCGGACATCTTCTACTCT | ENSG00000140941 | 15;93 | 43;18 | 19;9 |
| ***GABARAPL1*** | ATCGGAAAAAGGAAGGAGAAAAGATC | CAGGCACCCTGGCTTTTGG | ENSG00000139112 | 15;93 | 43;18 | 19;9 |
| ***SQSTM1*** | AATGCAACCTTCCACATCTGGC | CCCAGCCTGAAGTTATTGATTGTGC | ENSG00000126581 | 15;93 | 43;18 | 19;9 |
| ***DNM1L*** | CGACTCATTAAATCATATTTTCTCATTGTCAG | TGCATTACTGCCTTTGGCACACT | ENSG00000087470 | 15;93 | 43;18 | 19;9 |
| ***FIS1*** | CCTGGTGCGGAGCAAGTACAA | TCCTTGCTCCCTTTGGGCAG | ENSG00000214253 | 15;93 | 43;18 | 19;9 |
| ***OPA1*** | AAATACTTTAAGGCAACAACTTACAAATACTGAA | AGCAAAATCTTCCAATACCTCTTTAACATTTT | ENSG00000198836 | 15;93 | 43;18 | 19;9 |
| ***MFN1*** | CTGAGGATGATTGTTAGCTCCACG | CAGGCGAGCAAAAGTGGTAGC | ENSG00000171109 | 15;93 | 43;18 | 19;9 |

***Abbreviations****: RPLP0, ribosomal protein lateral stalk subunit P0; B2M, beta-2-microglobulin; PPIA, peptidylprolyl isomerase A; MYH7, myosin heavy chain 7; BNIP3, BCL2/Adenovirus E1B 19 kDa protein-interacting protein 3, BNIP3L, BCL2/Adenovirus E1B 19 kDa protein-interacting protein 3-like; FUNDC1, FUN14 domain-containing protein 1; PINK1,* *PTEN-induced putative kinase 1; OPTN, Optineurin; CALCOCO2, Calcium Binding And Coiled-Coil Domain 2; LC3B (MAP1LC3B), microtubule associated protein 1 light chain 3 beta; GABARAPL1, GABA(A) receptor-associated protein like 1; SQSTM1, sequestosome 1; DNM1L, dynamin-1-like protein; FIS1, Mitochondrial fission 1 protein; OPA1, Optic Atrophy 1; MFN1, Mitofusin 1.* *N^†^= number of samples control; COPD patients, N^††^= number of samples of NID-COPD; ID-COPD patients, N^†††^= number of samples of patients with low CRP; patients with high CRP included per analysis.*

**Western Blot**

This manuscript contains a high amount of Western Blot data. Since the number of samples exceeds the maximal capacity of our gels, we used several gels per targets. Groups were divided over different gels as much as possible. Background correction was performed by rolling ball technique embedded in AI600 software. Correction within gels was performed by PonceauS staining, and between gels was performed using Factor Correction software.

In 3 out of 4 runs, gels were loaded with additional samples, to obtain additional data for a different, unrelated, and elsewhere submitted hypothesis. For this hypothesis, other targets were run on these gels as well, which are not included in this manuscript. Membranes were cut after PonceauS staining to minimize the amount of sample necessary for obtaining the data.

Distribution of samples and protein loading reference lanes is depicted in Supplementary Figure 3. Corrected protein quantity was presented as a fold change relative to the control group. Measurements were excluded when signs of protein deterioration were found or when specific samples contained high or other-than-average background. Photographs shown here are original photos, but they are saturated for review purposes. Quantification was performed on unsaturated original photographs.

A summary of all detected bands, and their relative height, is depicted in Supplementary Figure 4. All original photos are included in figure Supplementary Figure 5-13.

**Run 1:** Membranes were stained with PonceauS and OXPHOS was blotted (Supplementary Figure 3-4, 5-6). Unheated samples were used on a 4-12% gel since this antibody is known to perform better on unheated samples. OXPHOS proteins from top to bottom are ATP5A, UQCR2, MT-COI, SDHB, and NDUFB8. OXPHOS exposure time was 5 minutes. Blot layout #1 was used.

**Run 2:** Membranes were stained with PonceauS and LC3B, FUNDC1, BNIP3L, SQSTM1, and DNM1L were blotted (Supplementary Figure 3-4, 7-9). Heated samples were used on a 12% gel. LC3B and FUNDC1 exposure time was 5 minutes. SQSTM1 exposure time was 3 minutes. BNIP3L and DNM1L exposure time was 1 minute. Blot layout #1 was used. FUNDC1 (Supplementary Figure 7) (upper band) was re-probed on LC3B membrane, LC3B is therefore still visible as the two lower bands. SQSTM1 (F) (lower band) was re-probed on for this study unrelated mmebrane, upper band is still visible on F but does not interfere with SQSTM1 band and was not quantified. For DNM1L, an a-specific band was detected at +/- 200 kDa.

**Run 3:** Membranes were stained with PonceauS and Parkin was blotted (Supplementary Figure 3-4, 10-11). Heated samples were used on a 4-12% gel. Blot layout #1 was used.

**Run 4:** Membranes were stained with PonceauS and BNIP3 and GABARAPL1 were blotted (Supplementary Figure 3-4, 12-13). Heated samples were used on a 12% gel. Blot layout #2 was used. Two bands were detected for BNIP3, which are also present in C2C12 *in vitro* muscle cells. In this *in vitro* model, both bands were proven to be specific with siRNA knock-down. We quantified bands both together and separate, and since these analyses showed similar patterns we included only the combined analysis in this manuscript.

**Supplementary Table 2: Primary antibodies for western blotting.** Target protein, supplier of antibody, product number, research identifier, and specific number of samples per analysis are depicted for each target.

| **Target** | **Supplier** | **Product nr.** | **Dilution** | **Research identifier** | **N**^†^ | **N**^††^ | **N**^†††^ |
| --- | --- | --- | --- | --- | --- | --- | --- |
| **OXPHOS** | MitoSciences | MS604 | 1:1,000 | RRID:AB_2629281 | 14;95 | 44;19 | 19;9 |
| **BNIP3** | Cell signaling | 3769 | 1:1,000 | RRID:AB_2259284 | 14;94 | 44;18 | 19;9 |
| **BNIP3L** | Cell signaling | 12396 | 1:1,000 | RRID:AB_2688036 | 14;94 | 44;18 | 19;9 |
| **FUNDC1** | Santa Cruz | sc-133597 | 1:500 | RRID:AB_10609242 | 14;94 | 44;18 | 19;9 |
| **Parkin** | Cell signaling | 4211 | 1:1,000 | RRID:AB_2159920 | 14;92 | 43;18 | 18;9 |
| **LC3B** | Cell signaling | 2775 | 1:1,000 | RRID:AB_915950 | 14;93 | 43;18 | 19;9 |
| **GABARAPL1** | Proteintech | 11010-1-AP | 1:500 | RRID:AB_2294415 | 14;94 | 44;18 | 19;9 |
| **SQSTM1** | Cell signaling | 5114 | 1:1,000 | RRID:AB_10624872 | 14;94 | 44;18 | 19;9 |
| **DNM1L** | Cell signaling | 8570 | 1:1,000 | RRID:AB_10950498 | 14;94 | 44;18 | 19;9 |

***Abbreviations****: OXPHOS, oxidative phosphorylation; BNIP3, BCL2/Adenovirus E1B 19 kDa protein-interacting protein 3, BNIP3L, BCL2/Adenovirus E1B 19 kDa protein-interacting protein 3-like; FUNDC1, FUN14 domain-containing protein 1; LC3B (MAP1LC3B), microtubule associated protein 1 light chain 3 beta; GABARAPL1, GABA(A) receptor-associated protein like 1; SQSTM1, sequestosome 1; DNM1L, dynamin-1-like protein. N^†^= number of samples control; COPD patients, N^††^= number of samples of NID-COPD; ID-COPD patients, N^†††^= number of samples of patients with low CRP; patients with high CRP included per analysis.*

Supplementary Figure 3: Blot layouts of western blot gels. Two different blot layouts were used for western blotting. Protein standard is a mixture of several sample lysates and is equally run through all gels.


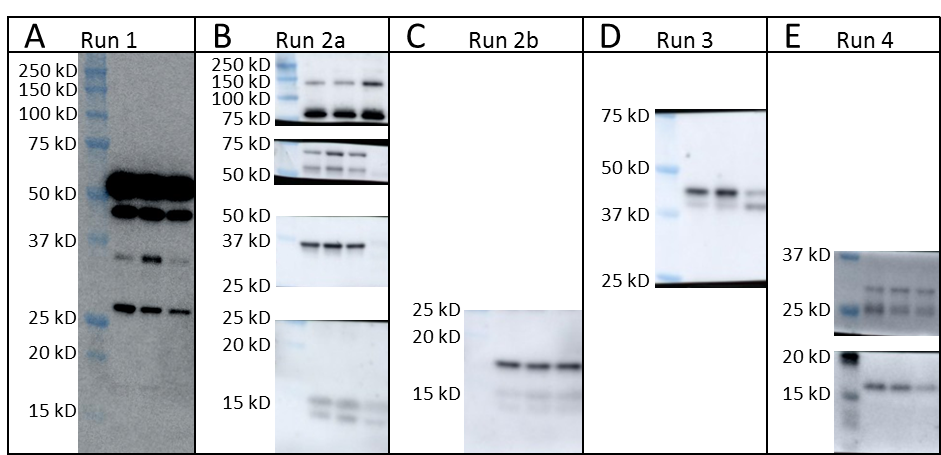


Supplementary Figure 4: Band heights and protein ladders. OXPHOS in run 1 (A); DNM1L, SQSTM1, BNIP3L, LC3B in run 2a (B); FUNDC1 in run 2b, re-probe of LC3B (C); Parkin in run 3 (D); BNIP3, GABARAPL1 in run 4 (E).


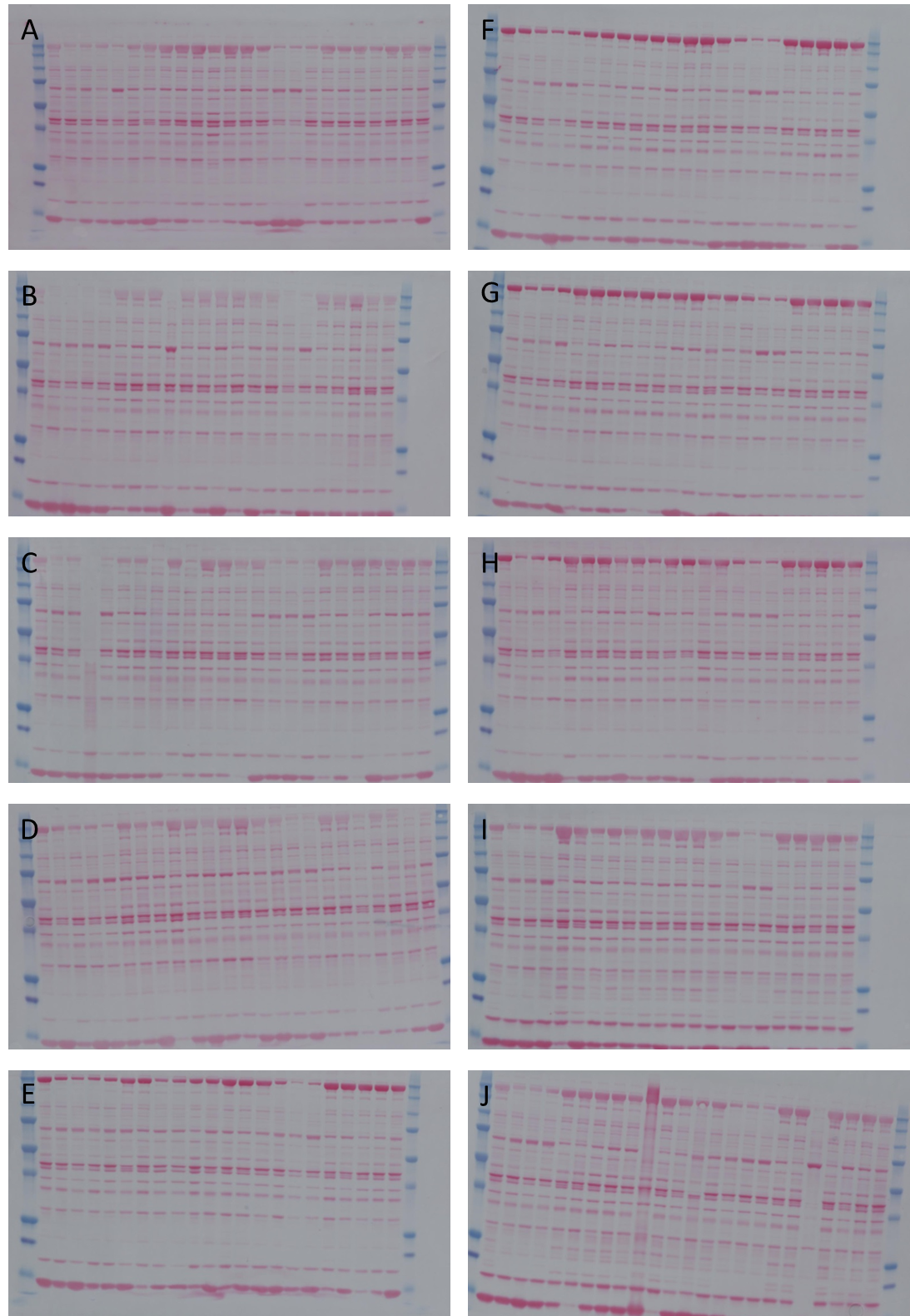


Supplementary Figure 5: PonceauS stains run 1. Gel 1-10 (A-J).


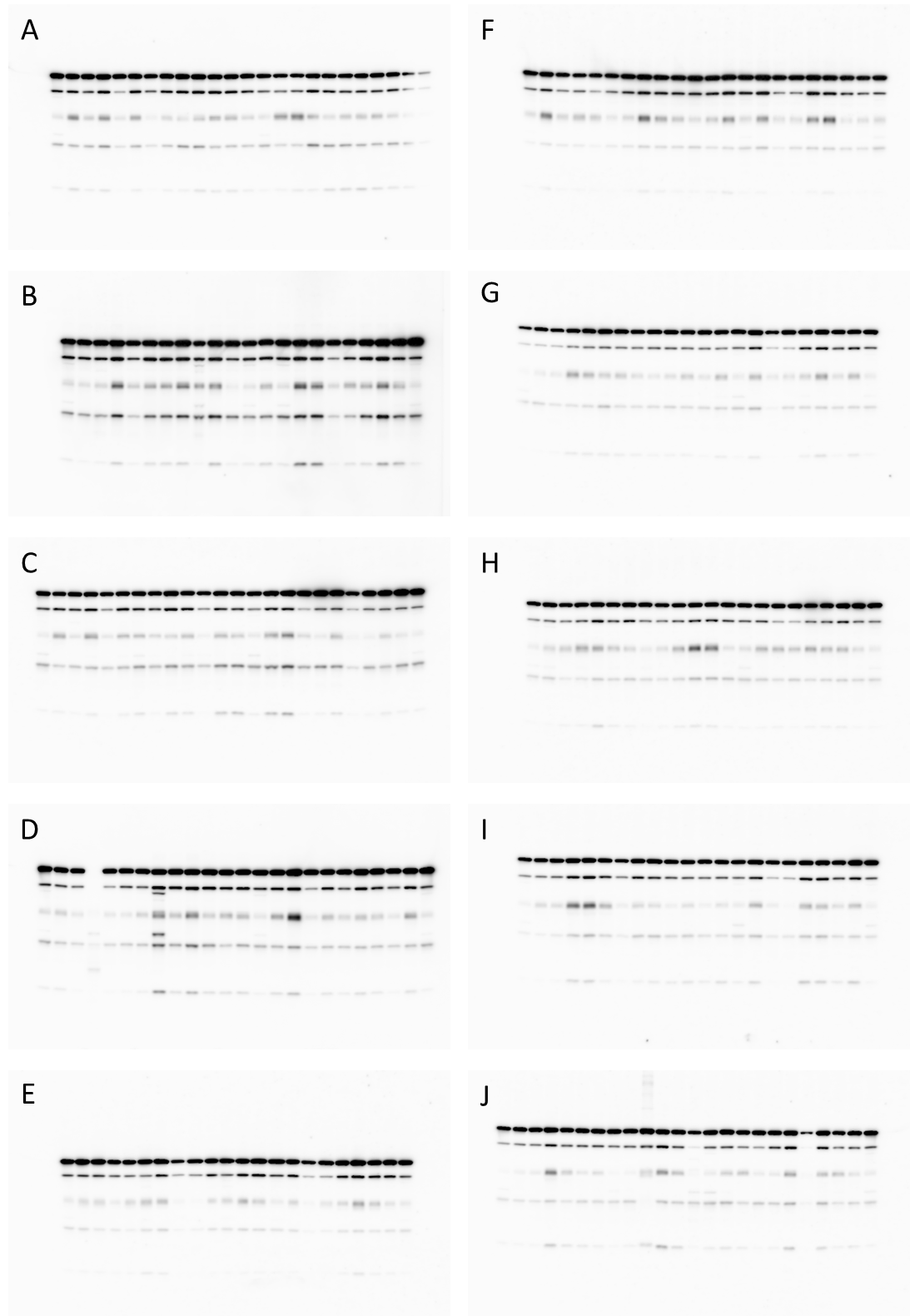


Supplementary Figure 6: blots run 1. OXPHOS blot 1-10 (A-J). No unspecific bands were detected.


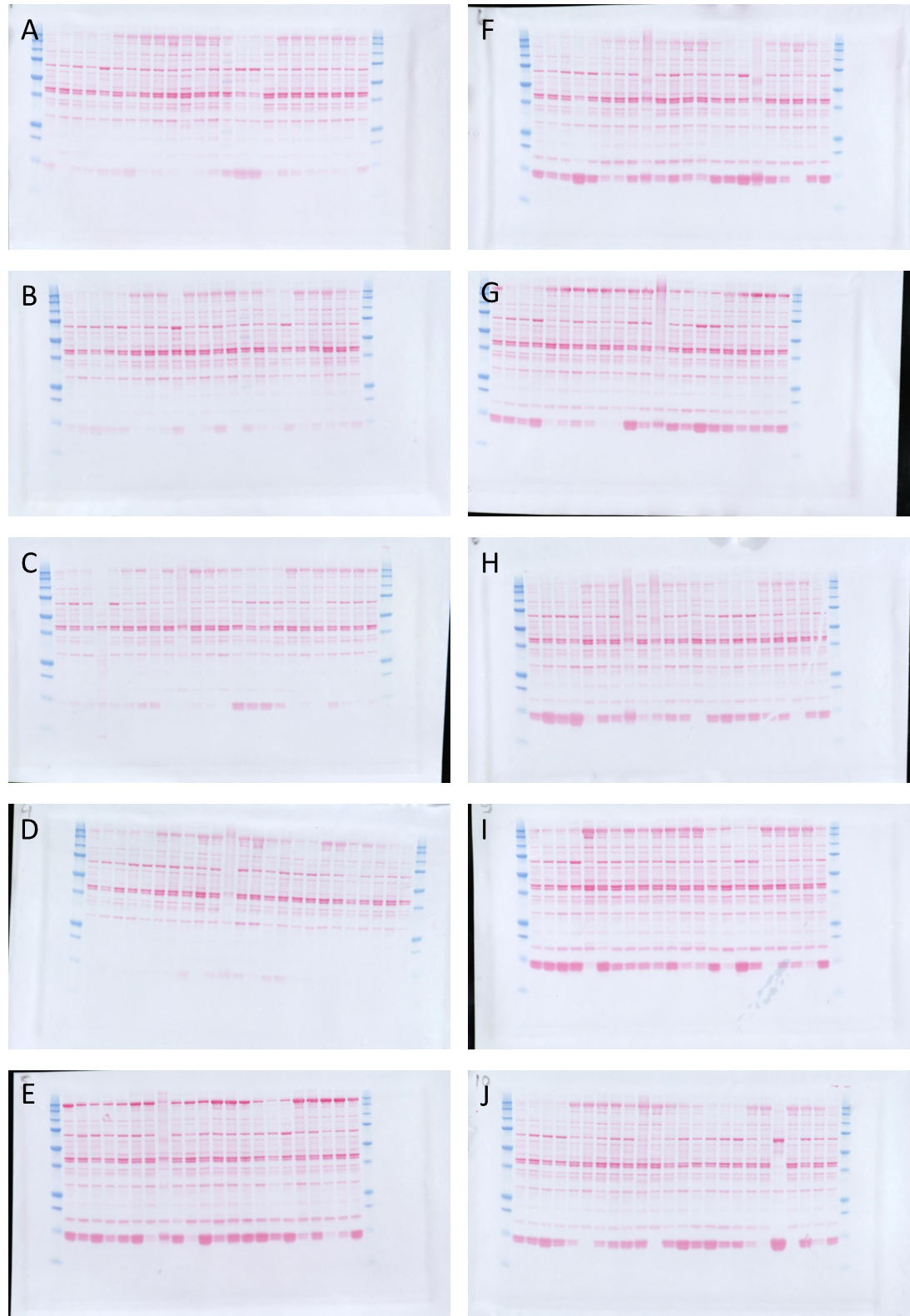


Supplementary Figure 7: PonceauS stains run 2. Gel 1-10 (A-J).

**
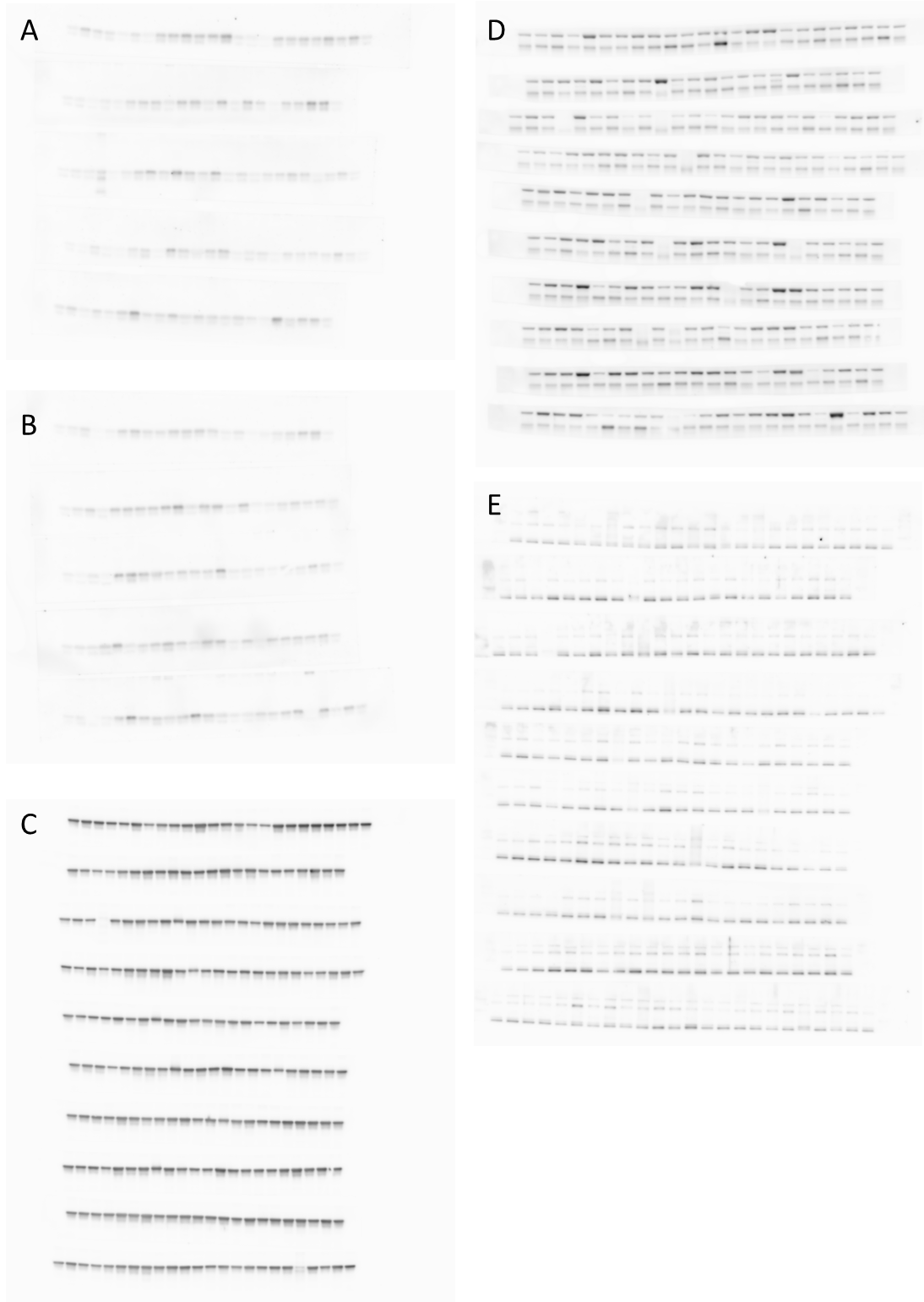
**

Supplementary Figure 8: Blots run 2a. LC3B 1-5 (A), LC3B 5-10 (B), BNIP3L 1-10 (C), SQSTM1 1-10 (F), DNM1L 1-10 (E).


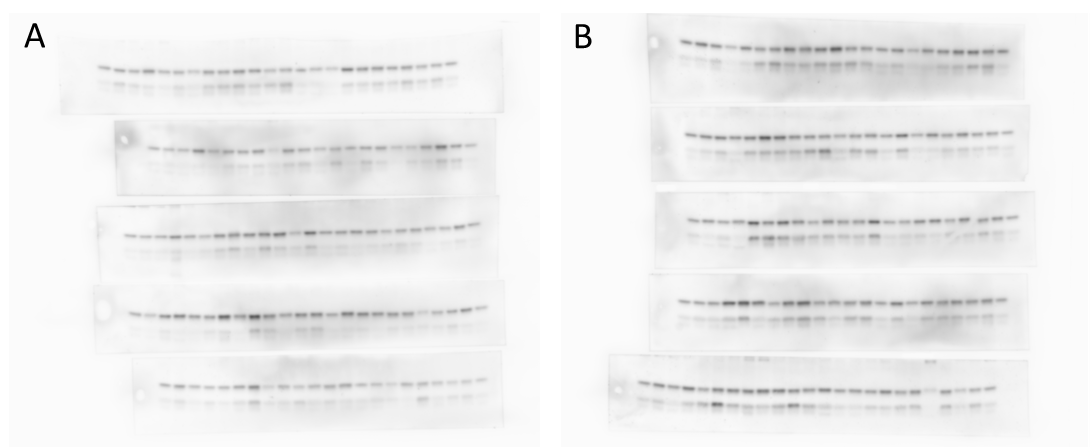


Supplementary Figure 9: Blots run 2b. FUNDC1 1-5 (A), FUNDC1 6-10 (B).


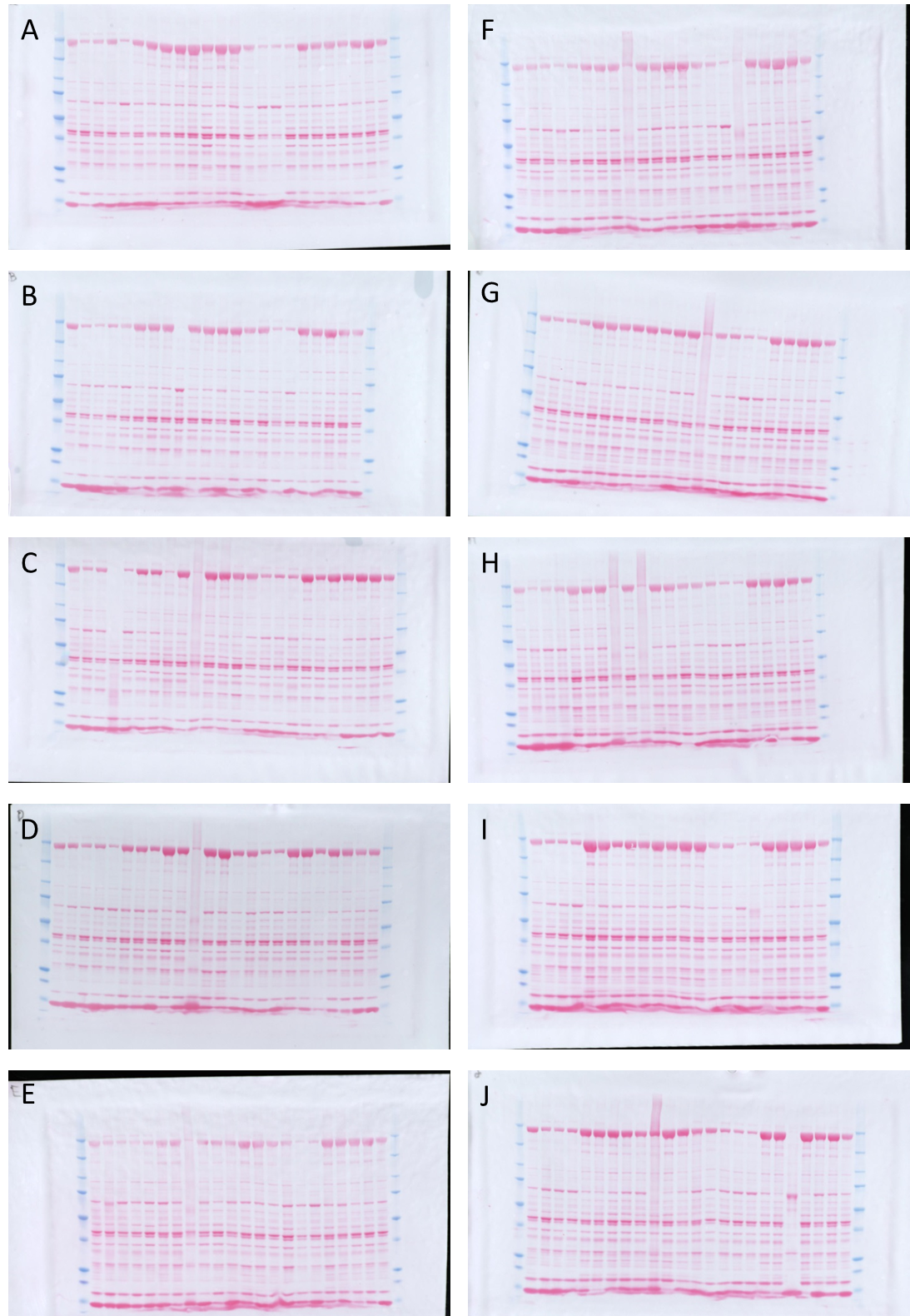


Supplementary Figure 10: PonceauS stains run 3. gel 1-10 (A-J).


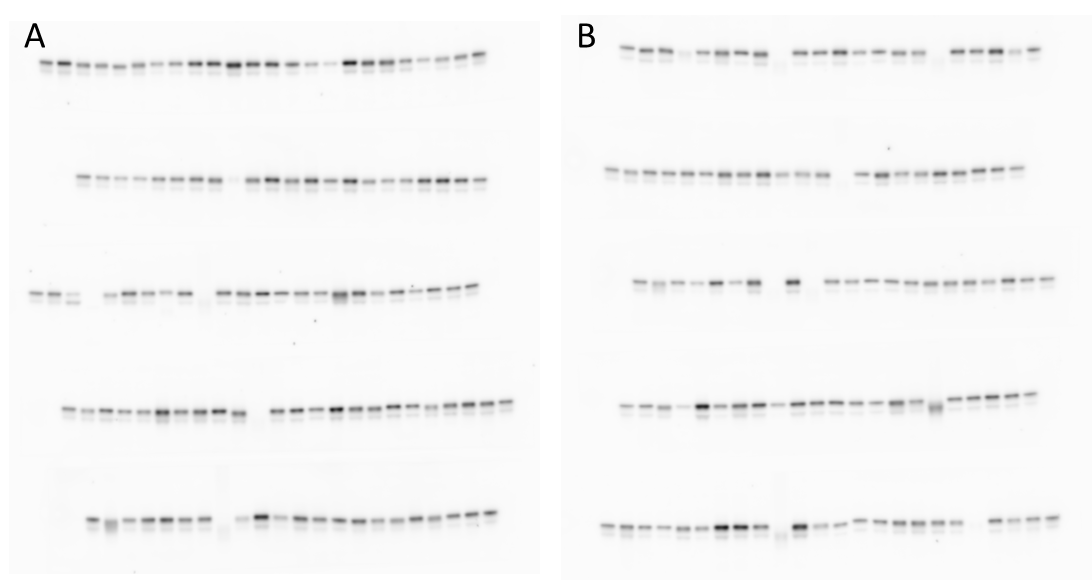


Supplementary Figure 11: Parkin 1-5 (A), Parkin 6-10 (B).


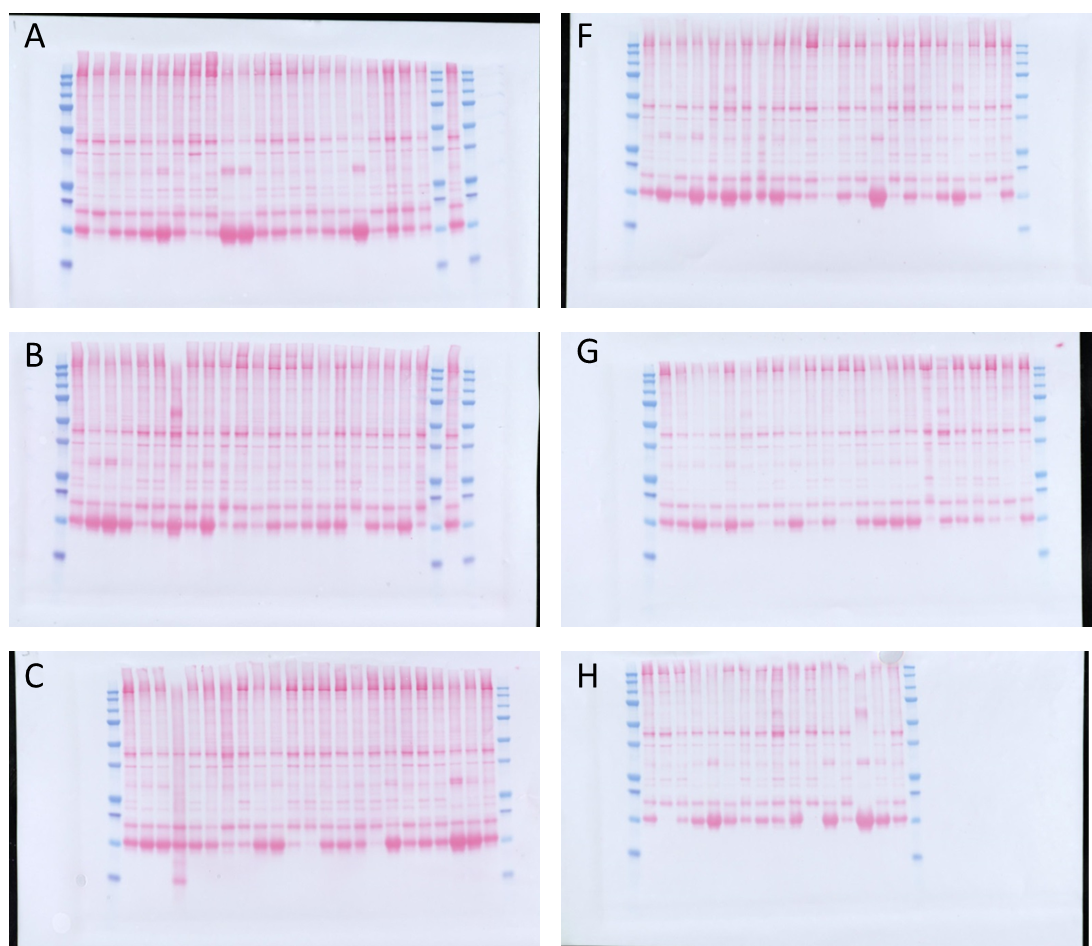


Supplementary Figure 12: PonceauS stains run 4. Gel 1-6 (A-H).


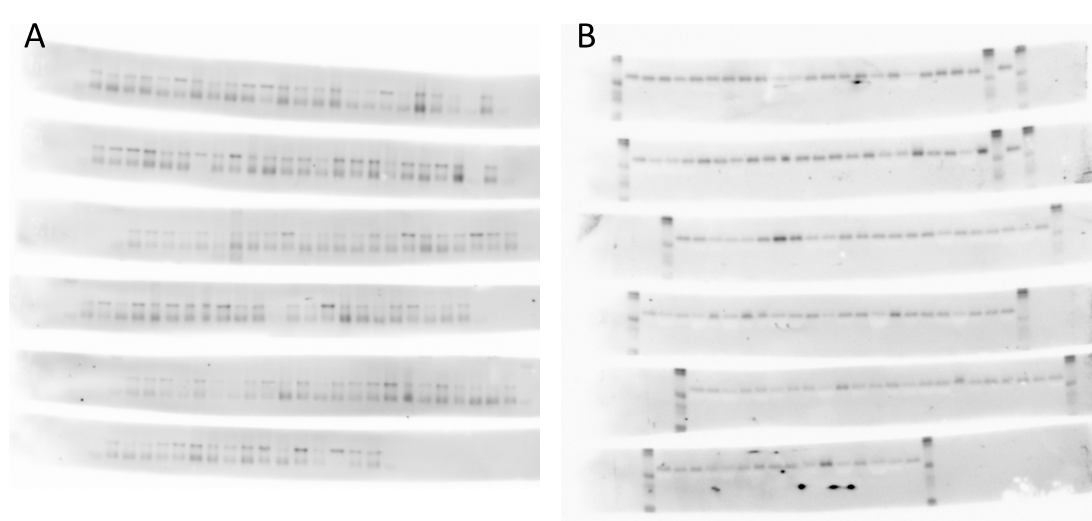


Supplementary Figure 13: BNIP3 (A), GABARAPL1 (B).
